# Improving 5-(hydroxymethyl)furfural (HMF) tolerance of *Pseudomonas taiwanensis* VLB120 by automated adaptive laboratory evolution (ALE)

**DOI:** 10.1016/j.mec.2024.e00235

**Published:** 2024-05-10

**Authors:** Thorsten Lechtenberg, Benedikt Wynands, Moritz-Fabian Müller, Tino Polen, Stephan Noack, Nick Wierckx

**Affiliations:** Institute of Bio- and Geosciences IBG-1: Biotechnology, Forschungszentrum Jülich, 52425 Jülich, Germany

**Keywords:** 5-(hydroxymethyl)furfural (HMF), Adaptive laboratory evolution (ALE), *Pseudomonas*, Aldehyde stress, MexT, MexEF-OprN

## Abstract

The aldehyde 5-(hydroxymethyl)furfural (HMF) is of great importance for a circular bioeconomy. It is a renewable platform chemical that can be converted into a range of useful compounds to replace petroleum-based products such as the green plastic monomer 2,5-furandicarboxylic acid (FDCA). However, it also exhibits microbial toxicity for example hindering the efficient biotechnological valorization of lignocellulosic hydrolysates. Thus, there is an urgent need for tolerance-improved organisms applicable to whole-cell biocatalysis. Here, we engineer an oxidation-deficient derivative of the naturally robust and emerging biotechnological workhorse *P. taiwanensis* VLB120 by robotics-assisted adaptive laboratory evolution (ALE). The deletion of HMF-oxidizing enzymes enabled for the first time evolution under constant selection pressure by the aldehyde, yielding strains with consistently improved growth characteristics in presence of the toxicant. Genome sequencing of evolved clones revealed loss-of function mutations in the LysR-type transcriptional regulator-encoding *mexT* preventing expression of the associated efflux pump *mexEF*-*oprN*. This knowledge allowed reverse engineering of strains with enhanced aldehyde tolerance, even in a background of active or overexpressed HMF oxidation machinery, demonstrating a synergistic effect of two distinct tolerance mechanisms.

## Highlights

• Robotics-assisted ALE in presence of HMF without interfering conversion.
• New and unforeseen mechanism of furanic aldehyde tolerance.
• Disruption of HMF-responsive regulator MexT prevents *mexEF*-*oprN* expression.
• Previously tolerance-enhanced BOX strains are further improved by *mexT* deletion.

## Graphical abstract

**Figure:**
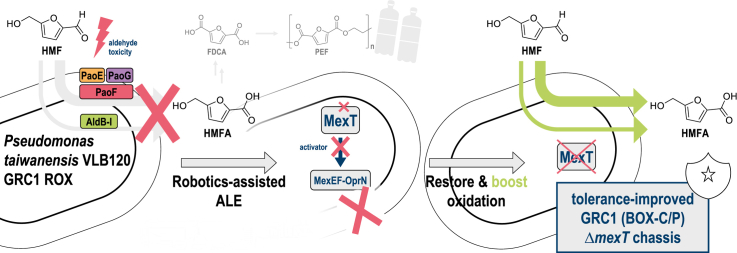

## 1 Introduction

Climate change is progressing rapidly and earth's fossil resources are dwindling fast thereby urgently suggesting a switch to more sustainable technologies like microbial biocatalysis with renewable carbon sources (de Lorenzo, 2022; Ragauskas et al., 2006). Particular potential as future feedstock is assigned to lignocellulosic biomass, non-edible plant waste material. However, rendering the locked sugars available to enzymes and microbes requires harsh pretreatment (Brethauer and Studer, 2015; Galbe and Wallberg, 2019; Prasad et al., 2023). This process typically involves mechanical grinding, heating and acidification resulting in the formation of unwanted by-products, so-called lignocellulose-derived microbial inhibitory compounds (LDMICs), e.g. phenolic aldehydes and acids (Ujor and Okonkwo, 2022). Especially under acidic conditions, pentoses and hexoses dehydrate to form the highly reactive and toxic furanic aldehydes furfural and 5-(hydroxymethyl)furfural (HMF) (Almeida et al., 2009; Jonsson and Martin, 2016). Their electrophilicity, caused by the polarity of the carbonyl function, makes them an easy target for all kinds of nucleophiles, among others, amino or thiol functionalities of proteins or DNA crosslinking the biological macromolecules and leading to malfunctions (Jayakody et al., 2018; Lee and Park, 2017; LoPachin and Gavin, 2014). As LDMICs prevent the efficient production of chemicals from biomass by non-tolerant microorganisms, they have to be removed in a laborious and costly manner prior to fermentation or avoided entirely through complex processes (Ujor and Okonkwo, 2022; Wang et al., 2020). Alternatively, this problem can be bypassed utilizing robust microbial species that display higher tolerance to the inhibitors (Jonsson et al., 2013).

Non-pathogenic soil-dwelling Pseudomonads like *P. taiwanensis* VLB120 can readily cope with various toxicants, most notably aromatics (Kohler et al., 2013; Volmer et al., 2014; Wynands et al., 2019). In addition, this emerging host for biotechnological applications convinces with its natural resilience to other compounds including aldehydes (Blombach et al., 2022; Wordofa and Kristensen, 2018). Recently, it was demonstrated that this tolerance mainly stems from the organism's ability to rapidly oxidize HMF to the less noxious carboxylic acid (Xu et al., 2020b). This process predominantly occurs in the periplasmic space avoiding damage inside the cell (Lechtenberg et al., 2024). Moreover, it was found that the tolerance could be increased by promoting oxidation through overexpression of the responsible enzymes in a genome-reduced chassis (GRC1) resulting in improved BOX (Boosted OXidation) strains (Lechtenberg et al., 2024; Wynands et al., 2019). However, conversion as tolerance mechanism has its limitations, especially when low cell densities face high aldehyde concentrations leading to prolonged lag phases (Heer and Sauer, 2008; López et al., 2021). This is especially relevant in the context of HMF biotransformation, where high initial substrate concentrations could simplify process operation. HMF hence plays a dual role, not only as an LDMIC but also as a promising platform chemical for a biobased chemical industry (Bozell and Petersen, 2010; Galkin and Ananikov, 2019; van Putten et al., 2013; Xu et al., 2020a). This opens up a wide array of potential biotechnological applications for tolerance-optimized bacteria the most important one being complete oxidation of HMF to 2,5-furandicarboxylic acid (FDCA), a renewable substitute for the plastic monomer terephtalic acid (Koopman et al., 2010; Pham et al., 2020; Troiano et al., 2020).

Adaptive laboratory evolution (ALE) provides an unbiased and natural selection process for advantageous mutations that can result in a diverse array of genetic variations. It is therefore a suitable method for microbial tolerance engineering, because this is typically a complex feature depending on numerous parameters, such as stressor-converting enzymes, energy supply, redox balance, membrane alteration, efflux pumps, and damage recovery through chaperones (Bitzenhofer et al., 2021; Dragosits and Mattanovich, 2013; Sandberg et al., 2019). Several of these factors (e.g. transporters and chaperones) are energy-dependent requiring tolerance evolution experiments to be precisely monitored and controlled (Hartl et al., 2011; Murakami et al., 2006). In order to preserve energy-consuming tolerance traits and avoid death caused by starvation in the toxic environment, cells should be permanently maintained under exponentially growing conditions, which can most effectively be achieved using a robotics platform (LaCroix et al., 2017). Automation brings further advantages including a higher passage frequency and an elevated number of possible replicates due to independency of human resources. Additionally, it leads to reduced fluctuation of inoculum density and permits real-time monitoring of culture parameters (Hirasawa and Maeda, 2023; Lee and Kim, 2020).

In this study, *P. taiwanensis* VLB120 GRC1 Δ*paoEFG* Δ*aldB-I* (GRC1 ROX, Reduced OXidation), deprived of aldehyde oxidation as its primary defense mechanism, was subjected to an automated ALE experiment. Continuous exposure of the bacterium to HMF stress yielded evolved strains with permanently increased tolerance towards the toxic aldehyde as well as to the related compound furfural. This performance advantage was attributed to loss-of-function mutations in the transcriptional regulator *mexT* identified by whole-genome sequencing and subsequent reverse engineering. Inactivation of *mexT* was shown to suppress *mexEF*-*oprN* expression, as a respective knockout of the efflux pump exhibited a similar phenotype. Furthermore, deletion of *mexT* was likewise beneficial in the wild type or BOX background proving an additive effect of two distinct tolerance mechanisms.

## 2 Results and discussion

### 2.1 Increasing HMF tolerance of oxidation-deficient GRC1 ROX by ALE

**Fig. 1:**
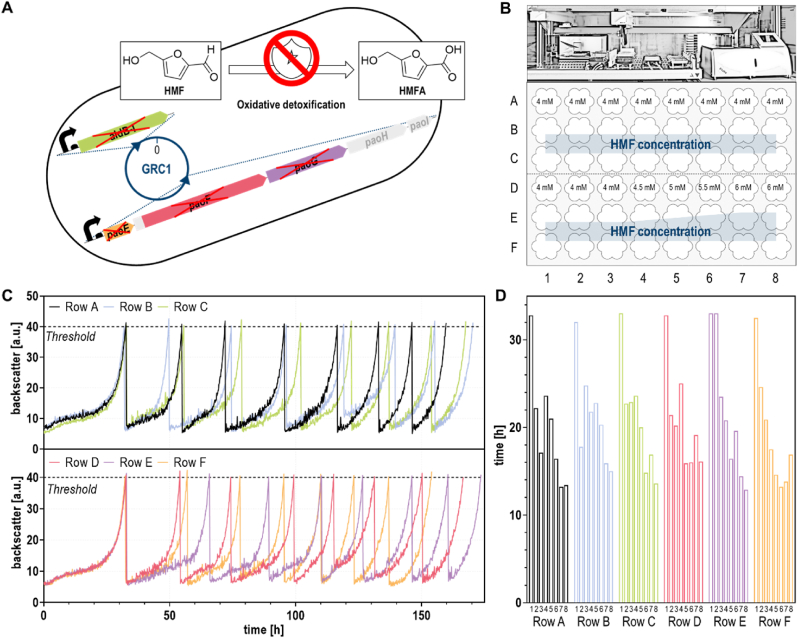
**ALE considerably accelerated growth of GRC1 ROX in the presence of HMF.** (**A**) Schematic representation of the employed oxidation-deficient starting strain, GRC1 ROX lacking PaoEFG and AldB-I. (**B**) Overview on the experimental setup. ALE was performed through automated serial passages in a 48-well FlowerPlate in a BioLector using MSM supplemented with 80 mM glycerol, 2 mM glucose and indicated HMF concentrations. Initial OD_600_ was set to 0.1 and subsequent batches were inoculated with 20 μL of the preceding culture. (**C**) Time course of the ALE experiment. Growth was monitored by scattered light intensities. Cultures were transferred to the next well, when a backscatter threshold of 40 was reached. (**D**) Elapsed time for each batch.

Hitherto, performing ALE experiments in the presence of aldehydes has been challenging due to the difficulty of maintaining a constant selection pressure. This is especially true if the investigated microorganism, as in the case of HMF oxidation by *P. taiwanensis* VLB120, promptly converts the stressor. The rapid oxidation often takes place in the span of about one microbial generation (Lechtenberg et al., 2024), and thus ALE would only lead to an increased tolerance towards the corresponding 5-(hydroxymethyl)-2-furoic acid (HMFA), rather than the far more toxic aldehyde. The recent deciphering of *Pseudomonas*’ enzymatic toolbox for oxidative HMF detoxification yielded the double deletion mutant GRC1 ROX*,* which lacks both the primary periplasmic aldehyde oxidoreductase PaoEFG as well as the supportive cytoplasmic dehydrogenase AldB-I thereby displaying drastically reduced HMF oxidation (Fig. 1A) (Lechtenberg et al., 2024). In an alternative pathway, this strain can still reduce HMF to 5-(hydroxymethyl)furfuryl alcohol (HMFOH), but this process is very slow in *Pseudomonas* and can be neglected (Lechtenberg et al., 2024). Therefore, GRC1 ROX was chosen for ALE, selecting for the activation of so far not expressed HMF-converting enzymes or the development of alternative tolerance traits. We hypothesized that this would help to uncover secondary aldehyde tolerance mechanisms by characterization of emerging mutations.

Due to severely reduced HMF tolerance in absence of oxidizing enzymes, growth of GRC1 ROX under various conditions was assessed in the BioLector to identify an optimal balance between high selection pressure and sufficient growth to enable ALE. Two-fold buffered minimal salt medium (MSM) containing 80 mM glycerol and 2 mM glucose as carbon sources, supplemented with HMF concentrations ranging from 4 to 5 mM, and inoculated to an OD_600_ of 0.1 was found to be suitable. These conditions ensured culture times of about one day spanning approximately six generations, pH stability, and exposure to the stressor throughout growth phase (Fig. S1). Using a dual carbon source is a common strategy for whole-cell biocatalytic conversion of HMF (Koopman et al., 2010; Lechtenberg et al., 2024; Pham et al., 2020). Glycerol constituted the main carbon source instead of glucose to prevent acidification from gluconate formation and a small amount of the sugar was added to reduce the lag phase. Six parallel ALE experiments were performed of which three were constantly exposed to 4 mM HMF and the other three to gradually increasing concentrations from 4 mM to 6 mM HMF (Fig. 1B). The threshold for automated reinoculation of the following batch was set to a backscatter value of 40 corresponding to late exponential growth phase (Fig. 1C, Fig. S1). While the initial cultures all grew almost identically, the second batch already reached the threshold considerably faster in five out of six cases (Fig. 1C). Despite some slower outliers, this trend continued until the end of the experiment with the final cultures requiring on average only 14.7 h to attain the target backscatter value. This was more than twice as fast as the initial cultures (Fig. 1D). No difference was observed between the two setups of constant or slightly increasing HMF concentrations. After a comparatively short ALE experiment of about one week with seven serial passages spanning approximately 40 generations, cultures with substantially improved HMF tolerance were obtained. For subsequent analysis, the cultures of well columns 7 and 8 were spread on LB agar and two single colonies isolated for each evolutionary line.

### 2.2 Characterization of evolved strains

**Fig. 2:**
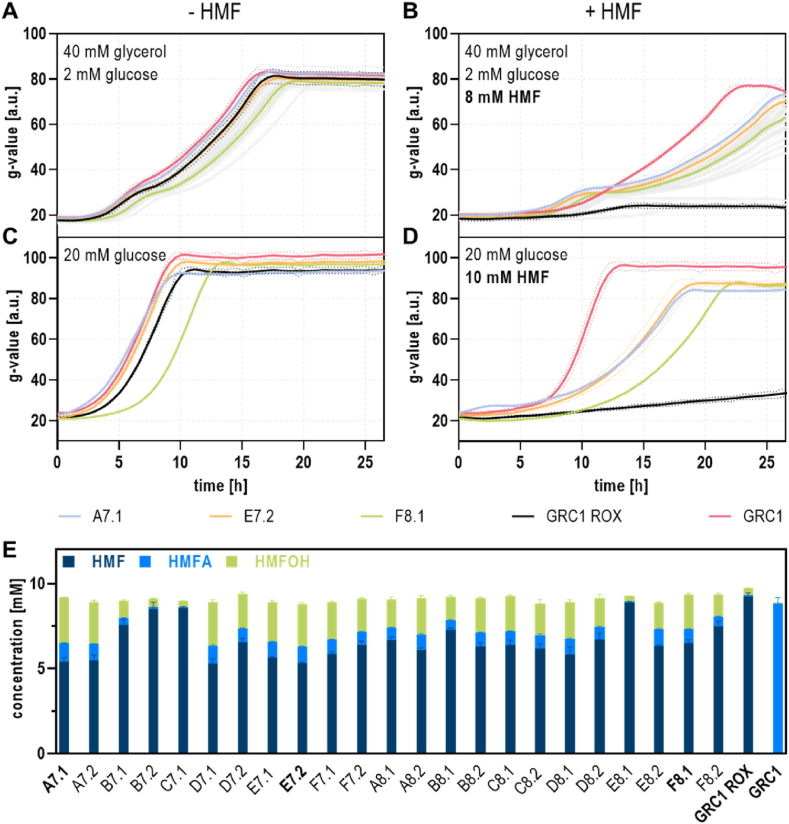
**Analysis of isolated clones from the ALE.** Growth comparison between all evolved clones (grey), the unevolved GRC1 ROX reference (black), and the oxidation-positive GRC1 (red) in absence (**A**/**C**) or presence of 8 mM (**B**) or 10 mM (**D**) HMF. Selected strains A7.1 (light blue), E7.2 (orange), and F8.1 (green) subsequently analyzed by whole-genome sequencing are highlighted by color. Cells were cultivated in a Growth Profiler in 96-well microtiter plates using two-fold buffered MSM supplemented with 40 mM glycerol and 2 mM glucose (**A**/**B**) or 20 mM glucose (**C**/**D**) as carbon sources. The initial OD_600_ of the cultures was set to 0.1. The growth curves result from a second-order smoothing to the mean values obtained from three replicates. The dots represent the standard deviation. (**E**) HPLC analysis of HMF tolerance assays with glycerol as principal carbon source (compare Fig. 2B, Fig. S2). Samples were taken after 25 h. The mean and standard deviation of three replicates is shown. (For interpretation of the references to color in this figure legend, the reader is referred to the Web version of this article.)

To verify whether the enhanced bacterial fitness was due to a temporal adaptation effect or beneficial genomic mutations, the evolved strains were tested for HMF tolerance in the Growth Profiler after undergoing an intermediate culture in aldehyde-free full medium. As expected, the generated strains showed similar behavior in the absence of stress compared to both the parental deletion mutant GRC1 ROX and the original GRC1 strain with intact aldehyde oxidation machinery (Fig. 2A). Slight differences for example in the case of strain F8.1 might be explained by the putative development of an energy-demanding tolerance trait (e.g. upregulation of an efflux pump) constituting a burden in a non-stressful environment (Wynands et al., 2019). When exposed to 8 mM HMF, most ALE-derived strains outperformed unevolved GRC1 ROX, which could not grow at all under these conditions (Fig. 2B, Fig. S2).

Next, the underlying physiological mechanisms behind the improved HMF tolerance were examined. Since the evolved strains did not surpass the parental control in absence of the stressor, it was unlikely that their fitness advantage was solely a result of adaptation to the utilized MSM or glycerol as a carbon source. This was verified by assessing their growth in presence of HMF using glucose as feedstock (Fig. 2C), where the strains showed the same tendencies as on glycerol. The evolved strains could grow on glucose with 10 mM HMF, whereas the starting strain GRC1 ROX could not (Fig. 2D). Restored oxidation ability or increased reductive detoxification was likewise excluded. *P. taiwanensis* VLB120 possesses a plethora of aldehyde oxidoreductases (Lechtenberg et al., 2024) leading to the hypothesis that, due to mutations, for example, in regulatory sequences, one or more of these enzymes could potentially replace the deleted principal HMF-converting enzymes PaoEFG and AldB-I. However, HPLC analysis at the end of the tolerance assays revealed no increased oxidation or reduction rates and the ratio between acid and alcohol formation remained almost constant (Fig. 2E). The observed differences in final acid and alcohol titers could rather be associated with minor growth variations. Specifically, faster-growing strains have higher average biomass density over the whole cultivation time, leading to higher volumetric conversions (Fig. 2E, Fig. S2). Thus, the observed improvement was likely a consequence of tolerance traits different from aldehyde conversion. In contrast to that, a previously furfural-tolerance-evolved *P. putida* KT2440, with functioning aldehyde oxidation, primarily featured a better conversion performance (Zou et al., 2022). For further insights, three selected evolved strains (A7.1, E7.2, F8.1) and the unevolved reference GRC1 ROX were subjected to whole-genome resequencing.

Genomic analysis uncovered only a few mutations compared to the reference sequence based on the published genome of *P. taiwanensis* VLB120 (GenBank accession number NC_022738) (Kohler et al., 2013), most of which were also present in the control strain. This narrowed down the list of potential factors for the increased HMF tolerance to a very limited number of promising candidates (Table 1), which was anticipated given the relatively short ALE with only seven or eight consecutive cultures. Since all analyzed evolved clones showed consistent behavior in presence of the stressor, similar genomic modifications were expected. The only gene found to be modified in all evolved strains, but not in the control, was *mexT* (PVLB_13900) encoding a LysR-type transcriptional regulator (www.pseudomonas.com (Winsor et al., 2016)). Strains A7.1 and F8.1 exhibited a point mutation leading to an amino acid exchange from glycine (G) to glutamate (E) at position 231, while E7.2 had a two-base-pair deletion resulting in a frameshift and a truncated protein version (Table 1). A premature stop codon in the *tig* (PVLB_08210) gene, which encodes a trigger factor involved in protein folding (Wu et al., 2022), was not considered critical, as it was exclusive to A7.1 (Table 1). To verify whether the alteration of *mexT* was responsible for the increased HMF tolerance of the evolved strains, reverse engineering of GRC1 ROX was performed. This was based on the mutation in strains A7.1 and F8.1, which was verified by PCR and Sanger sequencing.

**Table 1:** **Selected genomic loci affected by the ALE.** Abbreviations: nt, nucleotide; SNV, single nucleotide variant; del, deletion.

nt pos.	mutation	affected locus	frequency [%]			
			GRC1 ROX	A7.1	E7.2	F8.1
1,845,845	SNV_C_T (Q255*)	*tig* (PVLB_08210)	–	96.2	–	–
2,954,511	SNV_G_A (G231E)	*mexT* (PVLB_13900)	–	91.1	–	59.5
2,954,557	del_GG_-	*mexT* (PVLB_13900)	–	–	13.4	–

### 2.3 Reverse engineering

**Fig. 3:**
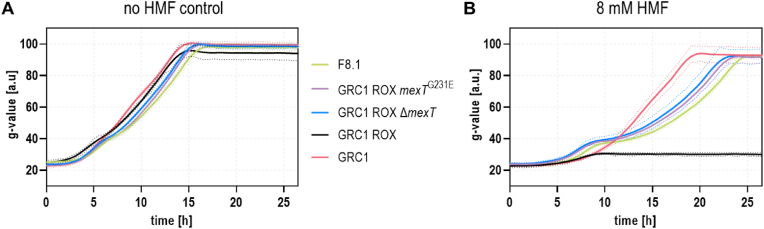
**Characterization of reverse-engineered GRC1 ROX mutants.** Two-fold buffered MSM supplemented with 40 mM glycerol and 2 mM glucose as carbon sources was inoculated with ALE strain F8.1 (green), GRC1 ROX *mexT*^G231E^ (purple), GRC1 ROX Δ*mexT* (dark blue), GRC1 ROX (black), and GRC1 (red) to an OD_600_ of 0.1. Cells were cultivated in a Growth Profiler in 96-well microtiter plates without HMF (**A**) and in presence of 8 mM HMF (**B**). The growth curves result from a second-order smoothing to the mean values obtained from three replicates. The dots represent the standard deviation. (For interpretation of the references to color in this figure legend, the reader is referred to the Web version of this article.)

The native *mexT* gene of GRC1 ROX was replaced by *mexT*^G231E^ using the homologous recombination-based I-SceI system (Martinez-Garcia and de Lorenzo, 2011) and the growth behavior of the resulting strain was analyzed. Similar to the evolved strains and in contrast to the unevolved progenitor, the reverse-engineered GRC1 ROX *mexT*^G231E^ mutant was able to grow in presence of 8 mM HMF, whereas growth was nearly unaffected without HMF (Fig. 3). Therefore, it could be concluded that the G231E amino acid exchange in MexT was responsible for the higher HMF tolerance of the ALE strains. The underlying SNV (G to A) likely represents a null mutation considering that E7.2 alternatively harbors a two-base-pair deletion generating a frameshift (Table 1). This hypothesis was confirmed by complete deletion of *mexT*, which led to the same phenotype as observed for the point mutation (Fig. 3B). Hence, we could prove that loss-of-function mutations in *mexT* were causal to the improved HMF tolerance.

**Fig. 4:**
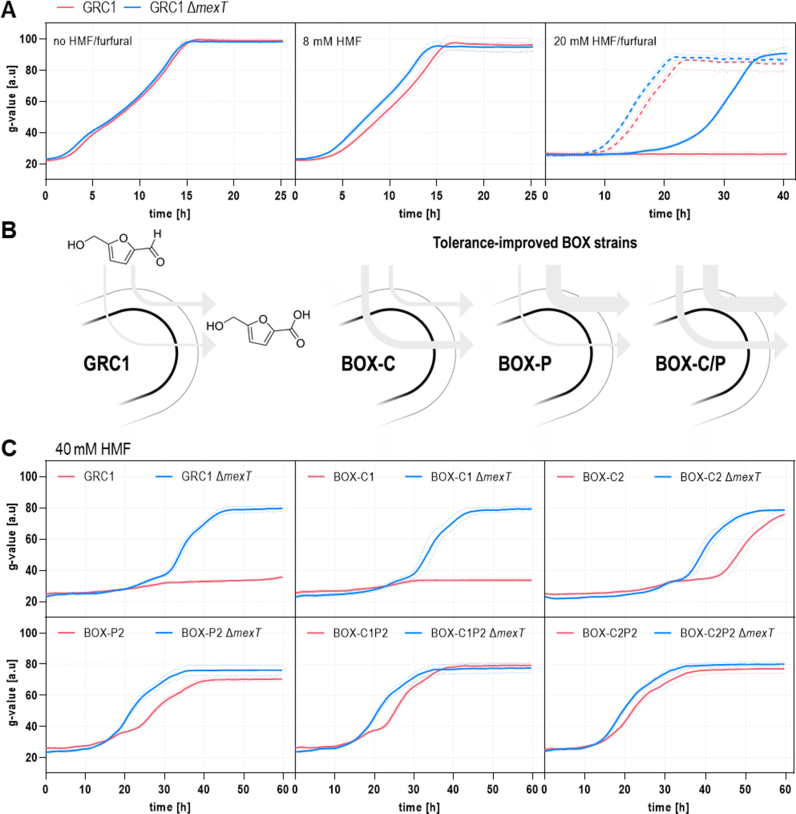
**Deletion of *mexT* also confers a fitness advantage in strains with intact or boosted aldehyde oxidation machinery when exposed to HMF or furfural.** (**A**) Growth of GRC1 (red) and GRC1 Δ*mexT* (blue) in absence of a stressor and in presence of 8 mM or 20 mM HMF (solid lines), or 20 mM furfural (dashed lines). (**B**) Overview of tolerance-improved BOX strains with increased HMF conversion in the cytoplasm, periplasm or both (Lechtenberg et al., 2024). (**C**) Growth of GRC1, the BOX derivatives (red) and the respective *mexT* deletion mutants (blue) in presence of 40 mM HMF. Control experiments without stressor addition are provided in Fig. S3. All experiments were carried out in two-fold buffered MSM supplemented with 40 mM glycerol and 2 mM glucose as carbon sources and an initial OD_600_ of 0.1. Cells were cultivated in a Growth Profiler in 96-well microtiter plates. The growth curves result from a second-order smoothing to the mean values obtained from three replicates. The dots represent the standard deviation. (For interpretation of the references to color in this figure legend, the reader is referred to the Web version of this article.)

Next, we sought to determine whether MexT inactivation would also have a beneficial effect in a strain with an intact aldehyde oxidation system, by deleting *mexT* in *P. taiwanensis* GRC1. Again, no difference between GRC1 and GRC1 Δ*mexT* was detected in absence of the stressor, but the deletion mutant showed a small growth advantage in the presence of 8 mM HMF, which became much more evident when the stressor concentration was increased to 20 mM (Fig. 4A). Under these conditions, the GRC1 strain was completely inhibited, whereas the Δ*mexT* mutant could still grow. A similar trend was observed when furfural was used as a toxicant (Fig. 4A). In accordance with previous determinations of EC_50_ values carried out with *P. putida* KT2440 (Jayakody et al., 2018), furfural had lower toxicity than HMF, and thus did not lead to a complete inhibition of the unmodified GRC1 at a concentration of 20 mM. Overall, these results confirmed that the MexT-related tolerance mechanism was compatible and even combinable with fast oxidation as *Pseudomonas*’ main line of defense against furanic aldehydes.

Recently, we reported the development of oxidation-optimized BOX strains, which showed increased HMF detoxification rates through different levels of overexpression of the central periplasmic aldehyde oxidoreductase *paoEFG* and the cytoplasmic aldehyde dehydrogenase *aldB-I* (Fig. 4B) (Lechtenberg et al., 2024). Even though these strains tolerate considerably higher aldehyde concentrations, this tolerance could still be further enhanced through additional deletion of *mexT*. A new series of BOX-C/P Δ*mexT* strains were grown in presence of 40 mM HMF (Fig. 4C). Under these harsh conditions, the *mexT* knockout variants consistently grew better than the respective unmodified control without exhibiting altered HMF oxidation rates (Fig. S4). However, the relative growth advantage was more pronounced as the oxidative capacity of the specific strain decreased, with the most substantial impact observed in the unmodified GRC1 and the only marginally improved BOX-C1 (Fig. 4C). This seems plausible, because these strains were exposed longer to high aldehyde stress, resembling the conditions during the ALE, not benefiting from improved fast periplasmic conversion. Interestingly, BOX-C2 Δ*mexT* had a longer lag phase than BOX-C1 Δ*mexT* although the parental BOX-C2 was considerably more tolerant than BOX-C1 pointing at a negative interference between the MexT-related tolerance mechanism and reinforced cytoplasmic HMF oxidation. That would also explain why the *mexT* deletion in BOX-C2P2 caused a weaker improvement than in BOX-P2 and BOX-C1P2 (Fig. 4C). On the other hand, it has also to be taken into account that BOX-C2P2 already showed a remarkable tolerance level *per se*, reducing the effect of further improvements. Considering this, it is even more notable that in the best-performing BOX strains an advancement by *mexT* deletion was still observed.

### 2.4 The benefit of *mexT* deletion lies in preventing *mexEF*-*oprN* expression

We attempted to further elucidate how inactivation of MexT mechanistically increased tolerance to furanic aldehydes. The protein is mainly characterized as transcriptional activator of the *mexEF*-*oprN* (PVLB_11800, PVLB_11795, and PVLB_11790) operon encoding a versatile RND-type efflux pump associated with antibiotic resistance in *Pseudomonas aeruginosa* (Juarez et al., 2018; Kohler et al., 1997, 1999). In *P. aeruginosa*, *mexEF*-*oprN* expression is increased upon exposure to aldehydes, such as cinnamaldehyde or citral (Tetard et al., 2019, 2021). However, the regulon is not restricted to the efflux pump and extends to at least 40 other targets, which is why MexT is generally more seen as redox-responsive regulator (Fargier et al., 2012; Tian et al., 2009). Recently the crystal structure of the regulatory domain of the *P. aeruginosa* homolog was elucidated, and the purified full-length protein employed in a DNase I footprinting assay to identify MexT binding sites (Kim et al., 2019). Comparing the established consensus sequence (ATCA(N)_7_CGAT) with the upstream region of *mexEF*-*oprN* in *P. taiwanensis* VLB120, we also found two putative MexT binding sites suggesting a similar regulatory network in the biotechnological workhorse (Fig. S5).

**Fig. 5:**
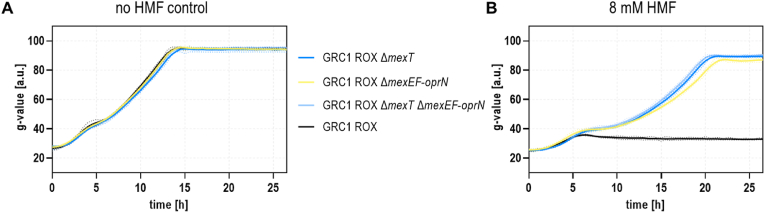
**The main effect of MexT inactivation occurring during ALE is a shutdown of the MexEF-OprN efflux pump.** Two-fold buffered MSM supplemented with 40 mM glycerol and 2 mM glucose as carbon sources was inoculated with GRC1 ROX Δ*mexT* (dark blue), GRC1 ROX Δ*mexEF-oprN* (yellow), GRC1 ROX Δ*mexT* Δ*mexEF-oprN* (light blue), and GRC1 ROX (black) to an OD_600_ of 0.1. Cells were cultivated in a Growth Profiler in 96-well microtiter plates without HMF (**A**) and in presence of 8 mM HMF (**B**). The growth curves result from a second-order smoothing to the mean values obtained from three replicates. The dots represent the standard deviation. (For interpretation of the references to color in this figure legend, the reader is referred to the Web version of this article.)

We initially hypothesized that furanic compounds like HMF or furfural might also be substrates of MexEF-OprN, which could thus contribute to increased tolerance by extrusion. This was supported by the fact that all three components, MexE, MexF, and OprN, were among the most upregulated, both at the transcriptional and translational levels, when *P. putida* KT2440 was exposed to thermochemical wastewater streams containing glycolaldehyde, HMF, and furfural as main toxicants (Jayakody et al., 2018). Moreover, the efflux pump was shown to be induced by formaldehyde and susceptibility to this aldehyde was elevated in a *mexE*-deficient background (Roca et al., 2008). However, this would have implied reverse regulation in *P. taiwanensis* VLB120 in comparison to *P. aeruginosa*, because *mexT* was actually disrupted during the ALE. Surprisingly, it was in fact the lack of activation of *mexEF*-*oprN* expression that was mostly responsible for the fitness advantage in the presence of HMF. This was confirmed by a GRC1 Δ*mexEF-oprN* deletion mutant, which, similar to GRC1 Δ*mexT*, could also grow in the presence of the stressor whereas no difference was observed in the control experiment without aldehyde addition (Fig. 5). In contrast to GRC1 Δ*mexT* or the double knockout GRC1 Δ*mexT* Δ*mexEF-oprN*, the growth rate appeared to be marginally reduced, especially towards the end of the growth period (Fig. 5B), indicating a slight impact of MexT beyond activation of the *mexEF*-*oprN* operon. This is certainly possible given the intricate MexT regulon.

An adverse effect of MexEF-OprN was not expected, but might be explained as follows: First, HMF could be an extremely strong effector molecule, resulting in MexT-mediated expression levels of *mexEF-oprN* which are high above a useful amount, only harming the cells through severe fitness costs of overexpressed transporter proteins (Du Toit, 2017). As RND-type efflux systems are proton antiporters, an increased efflux pump concentration likely disturbs proton potential gradient (Olivares et al., 2014). Moreover, it is possible that HMF is actually secreted by MexEF-OprN, but can diffuse back into the cell even faster, thereby generating a futile cycle of export and uptake, which again would only be a waste of energy. This kind of negative impact of a highly expressed efflux pump has already been discussed for the solvent efflux pump TtgGHI with regard to phenol tolerance (Wynands et al., 2019). Based on the role of MexT and MexEF-OprN in *P. aeruginosa,* a third possible explanation came up. It has been reported that MexEF-OprN could modulate quorum sensing through secretion of a signaling molecule belonging to the HAQ (4-hydroxy-2-alkylquinolines) family (Lamarche and Deziel, 2011). Although most publications focus on the influence of quorum sensing on virulence factors of *P. aeruginosa*, not relevant for *P. taiwanensis* VLB120, the bacterial signaling system could also be very important for tolerance mechanisms (Luong et al., 2014; Oshri et al., 2018). It is conceivable, for example, that the natural response of *P. taiwanensis* VLB120 to HMF stress is biofilm formation as protection against the toxicant. That would mean that the cells sense the toxic aldehyde (possibly through MexT) and retard growth until it is removed by oxidative conversion. As the GRC strains are deprived of the ability to form biofilms, this signaling cascade may be disturbed in our strains. Consequently, the bacteria keep up the precaution to not grow in presence of the stressor, although they could handle a moderate HMF concentration even without biofilm formation. The MexT inactivation might release this protective brake, but more experiments are necessary to gain further insights into the influence of quorum sensing on HMF tolerance.

## 3 Conclusion

Throughout evolution, microorganisms have encountered a diverse array of environmental conditions and effectively adapted to them. Nevertheless, employing even very robust bacteria such as *P. taiwanensis* VLB120 under industrial conditions represents a particular challenge (Blombach et al., 2022; Tan et al., 2022). We could improve tolerance of this promising biotechnological workhorse towards the increasingly important bio-based platform chemical HMF by a robotics-assisted ALE. This was enabled by taking two steps back: The rapid oxidation of furanic aldehydes to the less harmful acid derivatives was avoided by using the Reduced Oxidation mutant GRC1 ROX (Lechtenberg et al., 2024). We obtained considerably enhanced strains whose elevated tolerance was a result of a mechanism different from fast conversion, which is an interesting feature especially for biosynthetic endeavors where aldehydes occur as products or important intermediates (Bayer et al., 2017; Kunjapur and Prather, 2015; Narcross et al., 2016). The uncovered *mexT* disruption and associated MexEF-oprN repression were foundational for the increased HMF tolerance in a counter-intuitive fashion, considering that efflux pumps constitute a well-established mechanism for solvent tolerance and antibiotic resistance (Bitzenhofer et al., 2021; Henderson et al., 2021; Li et al., 2015). The *mexT* deletion could be synergistically combined with Boosted Oxidation in engineered BOX mutants (Lechtenberg et al., 2024) to further enhance HMF tolerance. This marks another step forward in the pursuit of generating an ideal chassis for whole-cell biocatalytic production of renewable plastic building block FDCA from HMF (Cui et al., 2023). With regard to a potential application of the improved strains as robust hosts for fermentative exploitation of lignocellulosic hydrolysates, future work will need to determine if LDMICs other than HMF and furfural, such as glycolaldehyde, or for example vanillin or *p*-hydroxybenzaldehyde derived from lignin depolimerization, are similarly well-endured (Hu et al., 2022; Jayakody et al., 2022). Additionally, it would be interesting to test how the engineered strains perform in the simultaneous presence of multiple stressors, some of which, unlike the furanic aldehydes HMF and furfural, may be better tolerated with a functional MexEF-OprN efflux pump.

## 4 Materials and methods

### 4.1 Strains and culture conditions

Chemicals used in this study were purchased from Sigma-Aldrich (St. Louis, MO, USA), Carl Roth (Karlsruhe, Germany), or Merck (Darmstadt, Germany) unless stated otherwise. Bacteria (refer to Table S1 for a list of all strains used in this study) were routinely cultivated in LB medium containing 10 g L^−1^ peptone, 5 g L^−1^ sodium chloride, and 5 g L^−1^ yeast extract or on solid LB with additional 15 g L^−1^ agar (Carl Roth, Karlsruhe, Germany). To isolate *Pseudomonas* strains following mating procedures irgasan (25 mg L^−1^) was supplemented. Growth experiments were performed using buffer-adjusted MSM (one-fold concentrations: 22.3 mM K_2_HPO_4_ and 13.6 mM NaH_2_PO_4_) according to Hartmans et al. (1989) with a mixture of glycerol (40 mM) and glucose (2 mM) as carbon sources unless stated otherwise. The use of glycerol prevented medium acidification due to gluconate formation resulting from *Pseudomonas*’ preference for metabolizing glucose via the Entner–Doudoroff pathway (Chavarría et al., 2013). This is important for all experiments involving HMF whose oxidative detoxification to HMFA can also lead to pH drop. Nevertheless, a low concentration of glucose was beneficial preventing an extensive lag phase. For the selection of genomic recombination events and plasmid maintenance, kanamycin sulfate (50 mg L^−1^) and gentamycin sulfate (20 mg L^−1^) were employed. *E. coli* was cultivated at 37 °C and *Pseudomonas* at 30 °C. Liquid cultures in shake flasks were incubated in a horizontal rotary shaker (Kuhner Shaker, Herzogenrath, Germany) with a humidity of 80%, a throw of 50 mm, and a frequency of 200 rpm. For cultures in 24-deepwell microplates (System Duetz), the frequency was raised to 300 rpm. High-throughput strain characterizations were performed with a Growth Profiler 960 (Enzyscreen, Heemstede, the Netherlands) allowing online growth measurements through image analysis of cultures in 96-well microtiter plates with transparent bottoms (CR1496dg). The resulting g-values (based on green pixel counts) correlate with the optical density of a cell culture, providing sufficient data for qualitative assessments. G-values were not converted into OD_600_, because calibrations hinge on cell shape and size, factors that may vary with different stressor concentrations, therefore requiring adjustments for each specific condition. Cultures in the 96-well format were conducted with a volume of 200 μL at 30 °C and 225 rpm shaking speed with an amplitude of 50 mm. The time gap between two photos used for growth analysis was 30 min. An overview of the growth parameters of all shown experiments is given in Table S4. Whole cell HMF bioconversion assays in 24-deepwell microplates (System Duetz) were carried out as described previously (Lechtenberg et al., 2024).

### 4.2 Robotics-assisted ALE

The ALE experiments were done on a Mini Pilot Plant covering a JANUS® liquid handler (PerkinElmer, Waltham MA, USA) and a BioLector I® (Beckmann Coulter Life Sciences, CA, United States). Repetitive batch cultivations were performed in 48-well FlowerPlates® (Beckmann Coulter Life Sciences, CA, United States) of the category BOH-1 (with optodes). All BioLector cultures were performed at 30 °C, shaking frequency of 1200 rpm, humidity of 85 % and oxygen-ratio of 20.95 % (air). Biomass (gain 20), pH and pO_2_ filters were used to monitor the growth progress. All media for the consecutive batches were stored sterile in a plate at room temperature on the deck of the platform and sealed with non-woven, gas-permeable sealing foil to minimize evaporation. The execution of the ALE was controlled by the Beckman RoboLection software, using the backscatter signal of the current batch as a trigger. When the culture reached a backscatter value of 40, a new batch was started so that the liquid handler pipetted 20 μL of the actual batch as inoculum and 780 μL of the specifically stored medium from the plate in an empty well.

### 4.3 Whole-genome sequencing

Genomic DNA for sequencing was isolated using the Monarch® Genomic DNA Purification Kit (New England Biolabs, Ipswich, MA, USA). DNA concentrations were determined employing a Qubit 2.0 fluorometer (Thermo Fisher Scientific, Waltham, MA, USA). Afterwards, 1 μg of DNA was used for library preparation with the NEBNext® Ultra™ II DNA Library Prep Kit for Illumina® (New England Biolabs, Ipswich, MA, USA). The library was subsequently evaluated by qPCR using the KAPA library quantification Kit (PEQLAB, Erlangen, Germany). After normalization for pooling, paired-end sequencing with a read length of 2 × 150 bases was performed on a MiSeq system (Illumina, San Diego, CA, USA). The sequencing output (base calls) were obtained as demultiplexed fastq files. The data was processed (e.g. trimming, mapping, coverage extraction) using the CLC Genomic Workbench software (QIAGEN Aarhus A/S, Aarhus, Denmark). Reads were mapped against an adapted version of the *P. taiwanensis* VLB120 genome that included the GRC modifications and the deletions of *paoEFG* and *aldB-I*. The significance of the identified mutations was manually assessed. Sequencing data are stored in the NCBI Sequence Read Archive under BioProject PRJNA1061370 with the accession numbers SAMN39267274 (A7.1), SAMN39267275 (E7.2), and SAMN39267276 (F8.1).

### 4.4 Plasmid cloning and strain engineering

Plasmids were constructed via Gibson assembly (Gibson et al., 2009) using NEBuilder HiFi DNA Assembly (New England Biolabs, Ipswich, MA, USA) and verified by Sanger sequencing. Primers were obtained as unmodified DNA oligonucleotides from Eurofins Genomics (Ebersberg, Germany). All oligonucleotides and plasmids used in this study are summarized in Table S2 and Table S3. DNA for cloning purposes was amplified with Q5 High-Fidelity Polymerase (New England Biolabs, Ipswich, MA, USA). Restriction enzymes were purchased from New England Biolabs. Plasmid DNA and PCR amplicons were purified with the Monarch® Plasmid Miniprep Kit and Monarch® PCR & DNA Cleanup Kit, respectively (New England Biolabs, Ipswich, MA, USA). *E. coli* was transformed with DNA assemblies and purified plasmids utilizing a standard heat shock protocol (New England Biolabs, Ipswich, MA, USA). Cloned plasmids, deletions, and substitutions, were verified by colony PCR using the OneTaq 2X Master Mix with Standard Buffer (New England Biolabs, Ipswich, MA, USA). For improved efficiency, the template cell material was lysed in alkaline polyethylene glycol as described by Chomczynski and Rymaszewski (2006). The I-SceI-based system developed by Martínez-García and de Lorenzo [59] enabled seamless genomic modifications and was utilized as described previously. For knockouts, the 500 bp upstream and downstream flanking regions of the target (TS1 and TS2) were cloned between the two I-SceI restriction sites of pSNW2 and the resulting plasmid was transferred from *E. coli* PIR2 to the intended *Pseudomonas* recipient strain through conjugation. For this, mating procedures were executed according to Wynands et al. (2018). Analogously, the single-base pair exchange was carried out with a plasmid having the mutated *mexT* sequence inserted between the TS-sites. Three random clones underwent transformation with I-SceI-encoding plasmid pSW-2 initiating the second homologous recombination event without the need for induction by 3-methylbenzoate. Correct clones were cured of pSW-2 by restreaking on non-selective medium, and re-analyzed by PCR ensuring a pure culture. The single-base pair exchange in *mexT* was verified by Sanger sequencing.

### 4.5 Analytical methods

Optical densities of cell suspensions were measured at a wavelength of 600 nm (OD_600_) with an Ultrospec 10 photometer (Biochrom, Cambridge, UK). Furanic compound concentrations were determined using a 1260 Infinity II HPLC system equipped with an InfinityLab Poroshell 120 EC-C18 column (3.0 × 150 mm, 2.7 μm) column and the corresponding InfinityLab Poroshell 120 EC-C18 (3.0 × 5 mm, 2.7 μm) guard column (all Agilent, Santa Clara, CA, USA). Chromatography was carried out at a temperature of 40 °C using potassium acetate buffer (10 mM, pH = 5.5, A) and acetonitrile (B) as mobile phases, with a flow rate of 0.8 mL min^−1^ for a duration of 7 min. The gradient method for elution is shown in Table 2. UV detection was performed at distinct wavelengths for each compound: HMF at 280 nm, HMFA at 250 nm, and HMFOH at 220 nm. Retention times were 2.44 min, 0.95 min, and 2.28 min for HMF, HMFA, and HMFOH respectively. Standards of each chemical were purchased from Biosynth (Staad, Switzerland) and used for quantification.

**Table 2:** Gradient method used for HPLC measurements.

time [min]	A [%]	B [%]
**0**	97	3
**1**	85	15
**3**	60	40
**4**	60	40
**6**	97	3
**7**	97	3

## Funding

This work was supported by the German 10.13039/501100002347Federal Ministry of Education and Research via the project NO-STRESS [grant number 031B0852A] and by the European Union's Horizon 2020 research and innovation program via the project UPLIFT [grant agreement number 953073].

## CRediT authorship contribution statement

**Thorsten Lechtenberg:** Writing – review & editing, Writing – original draft, Visualization, Validation, Investigation. **Benedikt Wynands:** Writing – review & editing, Supervision, Conceptualization. **Moritz-Fabian Müller:** Visualization, Validation, Investigation. **Tino Polen:** Resources, Investigation. **Stephan Noack:** Writing – review & editing, Resources, Methodology, Conceptualization. **Nick Wierckx:** Writing – review & editing, Supervision, Project administration, Funding acquisition, Conceptualization.

## Declaration of competing interest

The authors declare that they have no known competing financial interests or personal relationships that could have appeared to influence the work reported in this paper.

## Data Availability

Data will be made available on request.
