# Supplementary material for: Improving 5-(hydroxymethyl)furfural (HMF) tolerance of Pseudomonas taiwanensis VLB120 by automated adaptive laboratory evolution (ALE)
Source: Metab Eng Commun. 2024 May 10;18:e00235. doi: 10.1016/j.mec.2024.e00235 (PMC11144800; doi:10.1016/j.mec.2024.e00235)
Supplement: Multimedia component 1 [file mmc1.pdf]

Supporting Information

Improving 5-(hydroxymethyl)furfural (HMF) tolerance of *Pseudomonas taiwanensis* VLB120 by automated adaptive laboratory evolution (ALE)

Thorsten Lechtenberg[1], Benedikt Wynands[1], Moritz Fabian Müller[1], Tino Polen[1], Stephan Noack[1], and Nick Wierckx[1]

[1]Institute of Bio- and Geosciences IBG-1: Biotechnology, Forschungszentrum Jülich, 52425 Jülich, Germany

*Corresponding author:

Nick Wierckx, Institute of Bio- and Geosciences, IBG-1: Biotechnology, Forschungszentrum Jülich GmbH, 52425 Jülich, Germany, phone: +49 2461 61-85247

E-mail: n.wierckx@fz-juelich.de

Table of contents:

Table S1 page 2

Table S2 page 4

Table S3 page 5

Figure S1 page 5

Figure S2 page 6

Figure S3 page 7

Figure S4 page 8

Figure S5 page 9

Table S4 page 10

References page 13

Table S1: Bacterial strains used in this study.

strain	relevant characteristics	reference
E. coli		
PIR2	F⁻, Δ*lac169*, *rpoS(Am)*, *robA1*, *creC510*, *hsdR514*, *endA*, *recA1*, *uidA(ΔMluI)*::*pir*; host for *oriV*(R6K) vectors in low copy number	Thermo Fisher Scientific
HB101 pRK2013	Sm^R, *hsdR-M⁺*, *proA2*, *leuB6*, *thi-1*, *recA*; bears plasmid pRK2013	(Ditta et al., 1980)
DH5α pSW-2	DH5α bearing pSW-2	(Martinez-Garcia and de Lorenzo, 2011)
P. taiwanensis		
GRC1	genome-reduced chassis of *P. taiwanensis* VLB120, ΔpSTY, Δprophage1/2, Δprophage3, Δprophage4, Δflag1, Δflag2, Δlap1, Δlap2, Δlap3	(Wynands et al., 2019)
GRC1 ROX	GRC1 with Δ*paoE*, Δ*paoF*, Δ*paoG* , Δ*aldB-I*	(Lechtenberg et al., 2024)
ALE A7.1	GRC1 ROX after continuous exposure to 4 mM HMF, seven sequential cultures, evolution line A	this work
ALE E7.2	GRC1 ROX after exposure to increasing concentrations of HMF (4 – 6 mM), seven sequential cultures, evolution line E	this work
ALE F8.1	GRC1 ROX after exposure to increasing concentrations of HMF (4 – 6 mM), eight sequential cultures, evolution line F	this work
GRC1 ROX $mexT^{G231E}$	GRC1 ROX with $mexT^{G231E}$ (reverse-engineered)	this work MiKat #1018
GRC1 ROX Δ*mexT*	GRC1 ROX with Δ*mexT* (PVLB_13900)	this work MiKat #974
GRC1 Δ*mexT*	GRC1 with Δ*mexT* (PVLB_13900)	this work MiKat #953
BOX-C1	GRC1 with P_{aldB-I}::P_{14f_nRBS}; promoter of *aldB-I* exchanged to P_{14f}	(Lechtenberg et al., 2024)
BOX-C2	GRC1 with P_{aldB-I}::P_{14f_BCD2}; promoter of *aldB-I* exchanged to P_{14f} + BCD2	(Lechtenberg et al., 2024)
BOX-P2	GRC1 with $P_{paoEFGHI}$::P_{14f_nRBS}; promoter of *paoEFGHI* exchanged to P_{14f}	(Lechtenberg et al., 2024)
BOX-C1P2	GRC1 with $P_{paoEFGHI}$::P_{14f_nRBS}, P_{aldB-I}::P_{14f_nRBS}; promoter of *paoEFGHI* exchanged to P_{14f}, promoter of *aldB-I* exchanged to P_{14f}	(Lechtenberg et al., 2024)
BOX-C2P2	GRC1 with $P_{paoEFGHI}$::P_{14f_nRBS}, P_{aldB-I}::P_{14f_BCD2}; promoter of *paoEFGHI* exchanged to P_{14f}, promoter of *aldB-I* exchanged to P_{14f} + BCD2	(Lechtenberg et al., 2024)
BOX-C1 Δ*mexT*	BOX-C1 with Δ*mexT* (PVLB_13900)	this work MiKat #2118
BOX-C2 Δ*mexT*	BOX-C2 with Δ*mexT* (PVLB_13900)	this work MiKat #2116
BOX-P2 Δ*mexT*	BOX-P2 with Δ*mexT* (PVLB_13900)	this work MiKat #2117
BOX-C1P2 Δ*mexT*	BOX-C1P2 with Δ*mexT* (PVLB_13900)	this work MiKat #2120

BOX-C2P2 Δ*mexT*	BOX-C2P2 with Δ*mexT* (PVLB_13900)	this work<br>MiKat #2119
GRC1 ROX Δ*mexEF-oprN*	GRC1 ROX with Δ*mexEF-oprN* (PVLB_11790, PVLB_11795, PVLB_11800)	this work<br>MiKat #1137
GRC1 ROX Δ*mexT* Δ*mexEF-oprN*	GRC1 ROX with Δ*mexT* (PVLB_13900) and Δ*mexEF-oprN* (PVLB_11790, PVLB_11795, PVLB_11800)	this work<br>MiKat #1136

Table S2: Plasmids used in this study.

plasmid	relevant characteristics	assembly description	reference
pRK2013	Km^R, *oriV*(RK2/ColE1), mob^+ tra^+		(Figurski and Helinski, 1979)
pSW-2	Gm^R, *oriV*(RK2), mob^+, *xylS*, P_m → *I-sceI*		(Martinez-Garcia and de Lorenzo, 2011)
**pSNW2 and derivatives**			
pSNW2	Km^R, *oriV*(R6K), lacZα-MCS flanked by two I-SceI sites P_{14g} BCD2 → *msfgfp*		(Volke et al., 2020)
pSNW2-*mexT*-G231E	pSNW2 bearing flanking sequences of *mexT* surrounding the full mutated version $mexT^{G231E}$, delivery vector to introduce *mexT* (PVLB_13900) point mutation c.692G>A (p.G231E)	HiFi DNA assembly; TS1-*mexT*, $mexT^{G231E}$, and TS2-*mexT* were amplified from ALE7.1 using colony PCR and integrated into pSNW2 via EcoRI/SalI	this work #307
pSNW2-*mexT* (pSNW2_PVLB_13900_KO)	pSNW2 bearing flanking sequences of *mexT*, *mexT* deletion delivery vector	HiFi DNA assembly; TS1-*mexT* and TS2-*mexT* were amplified from VLB120 gDNA and integrated into pSNW2 via EcoRI/SalI	this work #291
pSNW2-*mexEF-oprN* (pSNW2_PVLB_11790-11800_KO)	pSNW2 bearing flanking sequences of *mexEF-oprN*, *mexEF-oprN* deletion delivery vector	HiFi DNA assembly; TS1-*mexEF-oprN* and TS2-*mexEF-oprN* were amplified from VLB120 gDNA and integrated into pSNW2 via EcoRI/SalI	this work #360

Table S3: Oligonucleotides (name, sequence, and description) used as PCR primers for cloning procedures. Lower case letters indicate overhangs, letters representing the binding sequence are capitalized, customly added spacers are italicized, and restriction sites are underlined. Oligonucleotides used for diagnostic PCRs and sequencing reactions are not included.

name	Sequence (5' → 3')	description
**TL_191**	agggataacagggtaatctgATACCGGATGCTCGTTGG	TS1-*mexT* forward primer
**TL_192**	ccccaaggctCGGTGCGCTTCACCTAAAG	TS1-*mexT* reverse primer
**TL_193**	aagcgcaccgAGCCTTGGGGCAGACGGG	TS2-*mexT* forward primer
**TL_194**	gaagcttgcatgcctgcaggATTCCACAGCCAGAGAGTGCG	TS2-*mexT* reverse primer
**TL_241**	agggataacagggtaatctgGAGAGCGGTGAGACCCATTTG	TS1-*mexEF-oprN* forward primer
**TL_242**	agtcgttgaaGGAGAACTCCGCCAATGTATTAG	TS1- *mexEF-oprN* reverse primer
**TL_243**	ggagttctccTTCAACGACTCCTTTGGTTG	TS2- *mexEF-oprN* forward primer
**TL_244**	gaagcttgcatgcctgcaggGTCATGCTCCAAATCTTG	TS2- *mexEF-oprN* reverse primer

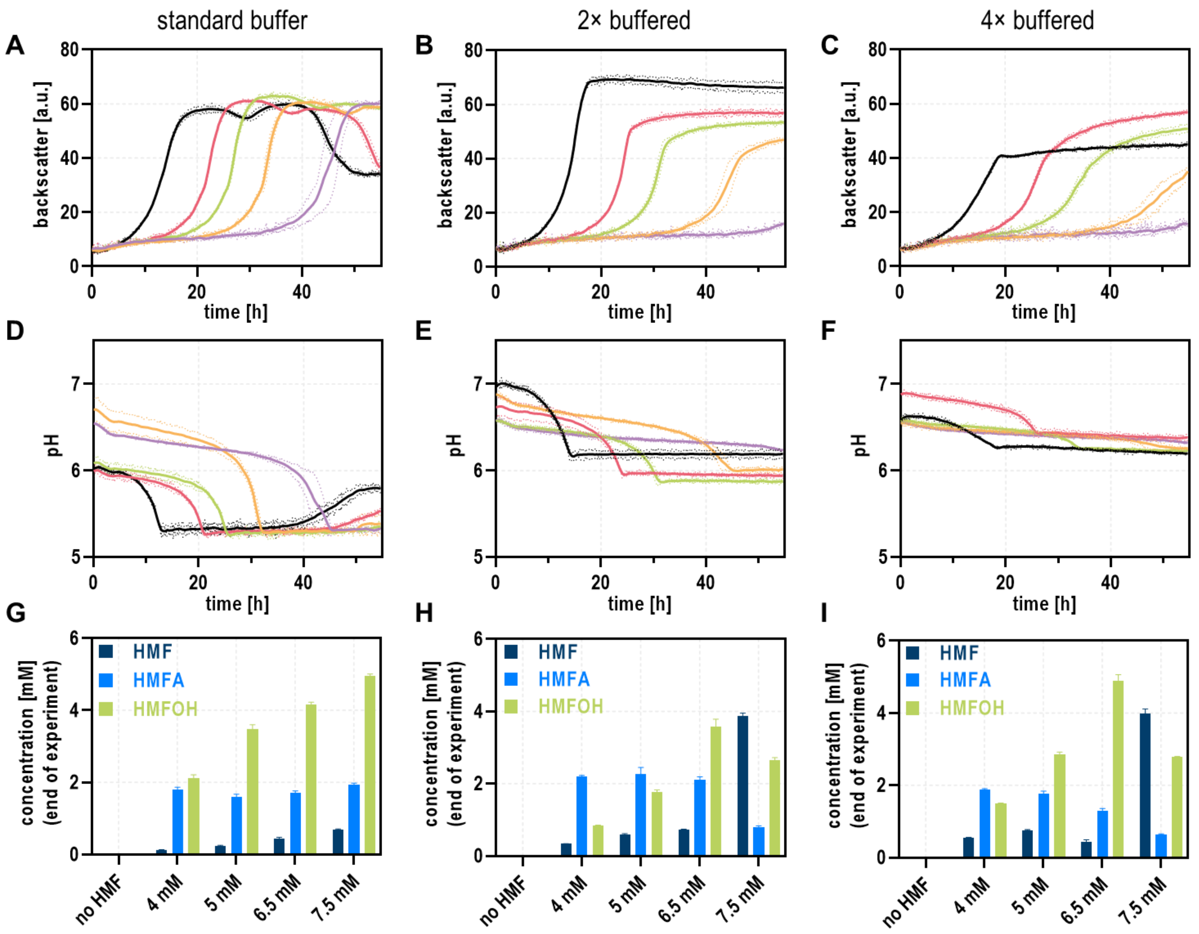

Figure S1: Evaluation of optimal culture conditions for an ALE with GRC1 ROX. All experiments were carried out in a 48-well FlowerPlate in a BioLector using MSM supplemented with 80 mM glycerol, 2 mM glucose, and varying concentrations of HMF (black: no HMF, red: 4 mM, green: 5 mM, orange: 6.5 mM, purple: 7.5 mM). Three buffer concentrations were tested: standard ((**A**), (**D**), (**G**)); two-fold ((**B**), (**E**), (**H**)); and four-fold ((**C**), (**F**), (**I**)). (**A**)-(**C**) Cell growth monitored by scattered light intensities. (**D**)-(**F**) pH values of the respective cultures. Graphs result from a second-order smoothing to the mean values obtained from three replicates. The dots represent the standard deviation. (**G**)-(**I**) HPLC analysis of final (55 h) HMF concentrations. The mean and standard deviation of three replicates is shown.

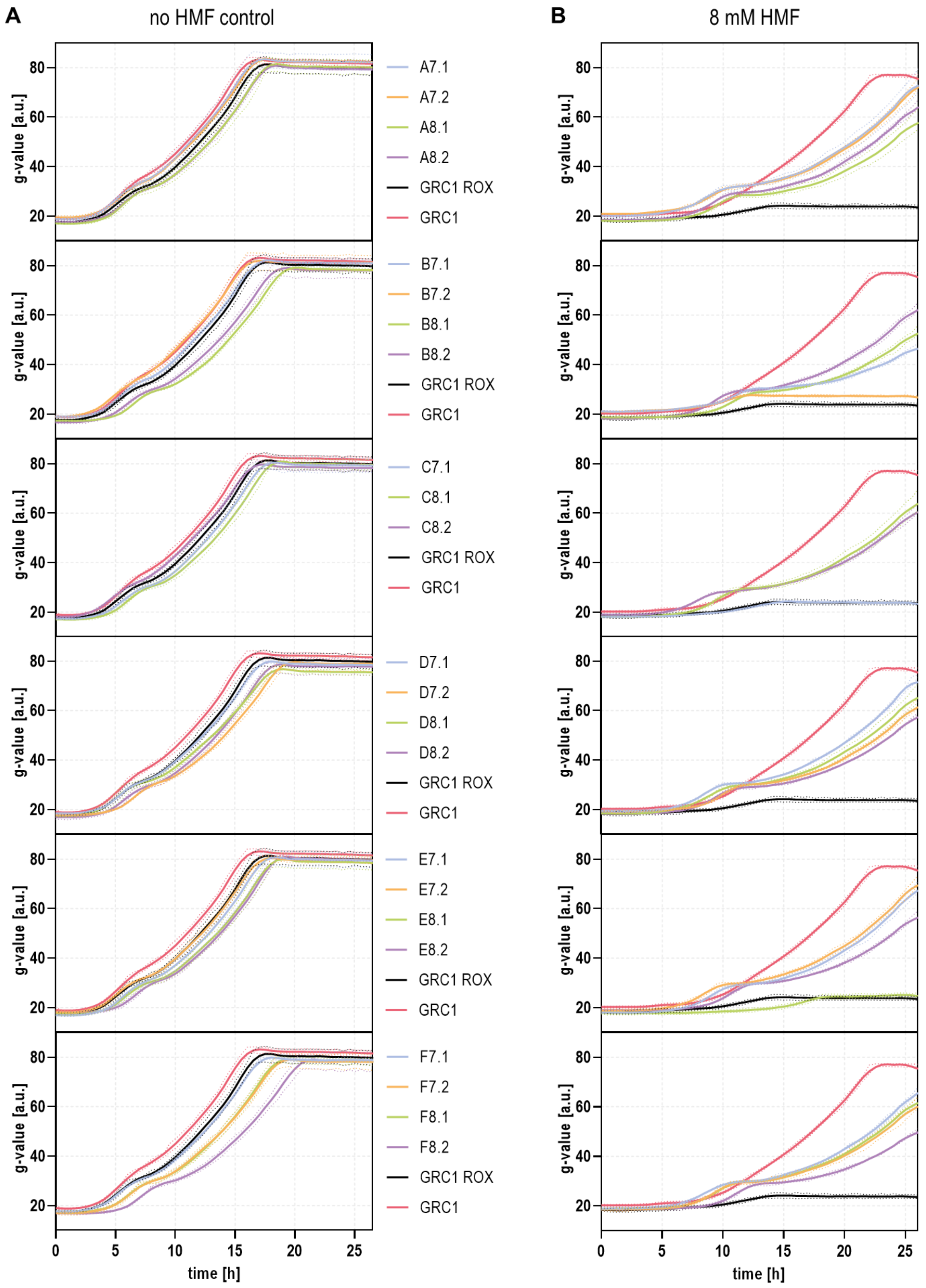

Figure S2: Analysis of all isolated clones from the ALE. Two-fold buffered MSM supplemented with 40 mM glycerol and 2 mM glucose as carbon sources was inoculated with the evolved strains (see legend for color coding), GRC1 ROX (black), and GRC1 (red) to an OD_{600} of 0.1. Cells were cultivated in a Growth Profiler in 96-well microtiter plates. The growth curves result from a second-order smoothing to the mean values obtained from three replicates. The dots represent the standard deviation. (**A**) No HMF added. (**B**) 8 mM HMF added. Because the preculture of clone C7.2 did not grow the respective isolate was not tested.

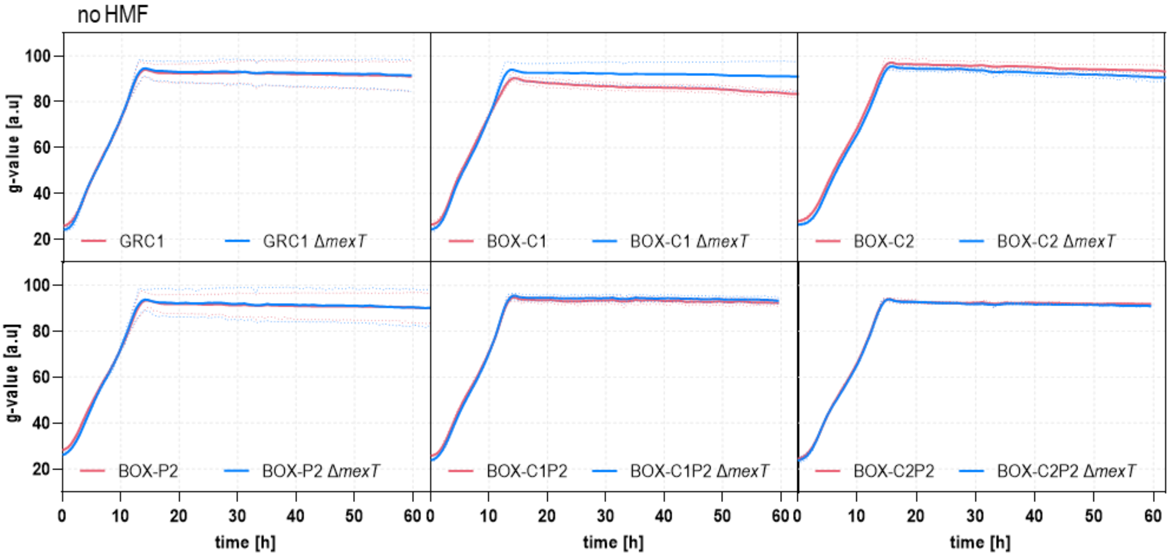

Figure S3: *MexT* deletion also confers a fitness advantage in strains with intact aldehyde oxidation machinery including the oxidation-optimized BOX strains (control experiments). Two-fold buffered MSM supplemented with 40 mM glycerol and 2 mM glucose as carbon sources in absence of HMF was inoculated with GRC1 or BOX derivatives (red) and the respective *mexT* deletion mutant (blue) to an OD_{600} of 0.1. Cells were cultivated in a Growth Profiler in 96-well microtiter plates. The growth curves result from a second-order smoothing to the mean values obtained from three replicates. The dots represent the standard deviation.

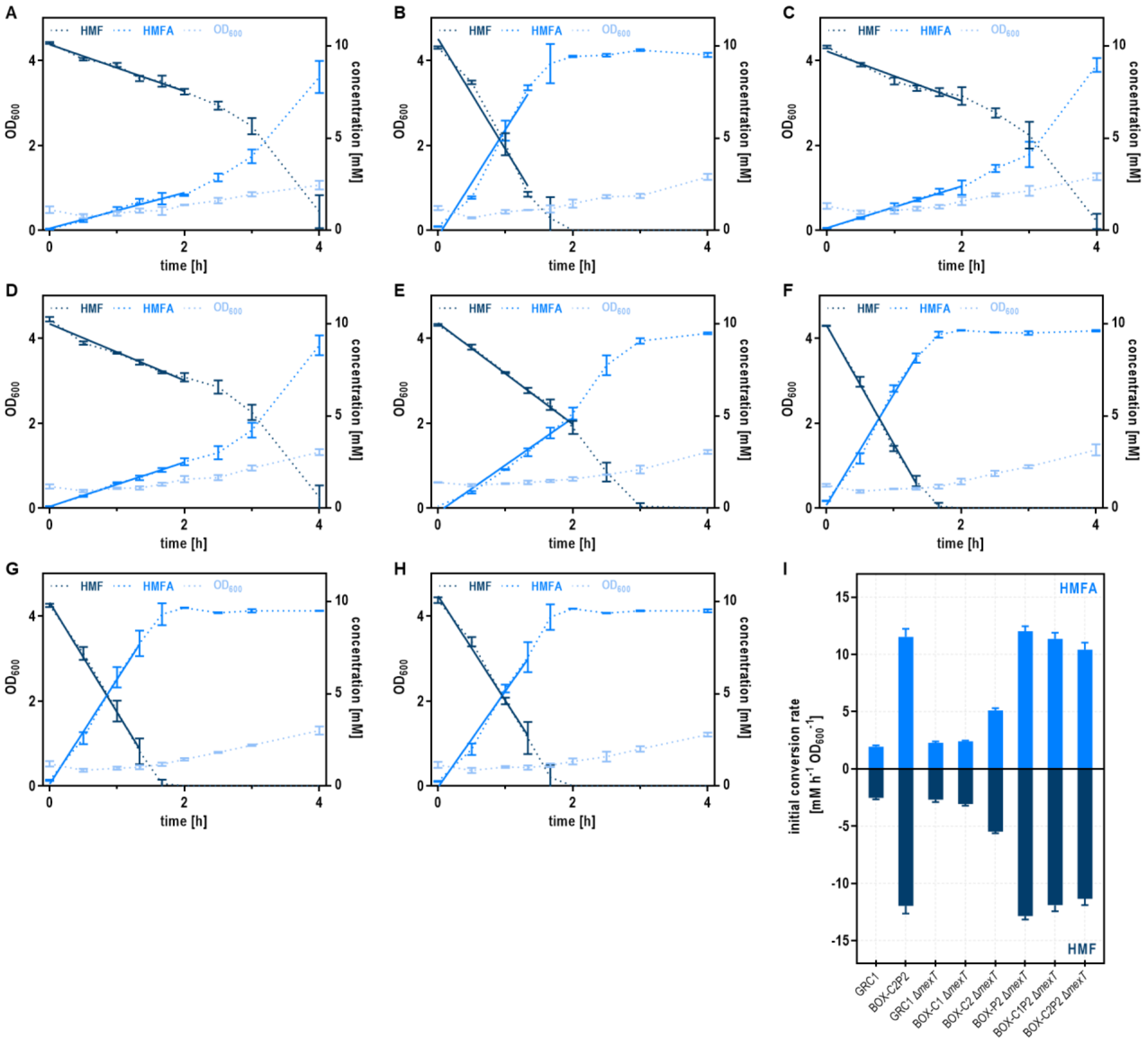

Figure S4: HMF conversion assays in 24-deepwell microplates (two-fold buffered MSM with 40 mM glycerol, 2 mM glucose, and 10 mM HMF) using whole-cells of GRC1 and derived BOX strains (increased expression of *paoEFG* and *aldB-I*) with deletion of *mexT*. For the determination of initial HMF depletion and HMFA formation rates a linear fit covering the first 2 h of each experiment was performed (shown as solid line). In cases HMF was fully converted faster this period was shortened accordingly. The OD_{600} was considered constant during that time and equaled the starting conditions. For each measurement, error bars represent the mean ± standard deviation of triplicates. (**A**) GRC1. (**B**) BOX-C2P2. (**C**) GRC1 Δ*mexT*. (**D**) BOX-C1 Δ*mexT*. (**E**) BOX-C2 Δ*mexT*. (**F**) BOX-P2 Δ*mexT*. (**G**) BOX-C1P2 Δ*mexT*. (**H**) BOX-C2P2 Δ*mexT*. (**I**) Initial HMF depletion and HMFA formation rates of all shown experiments. The error bars correspond to the standard error of the slope of the linear regression. *MexT* deletion does not alter HMF-oxidation properties of examined strains.

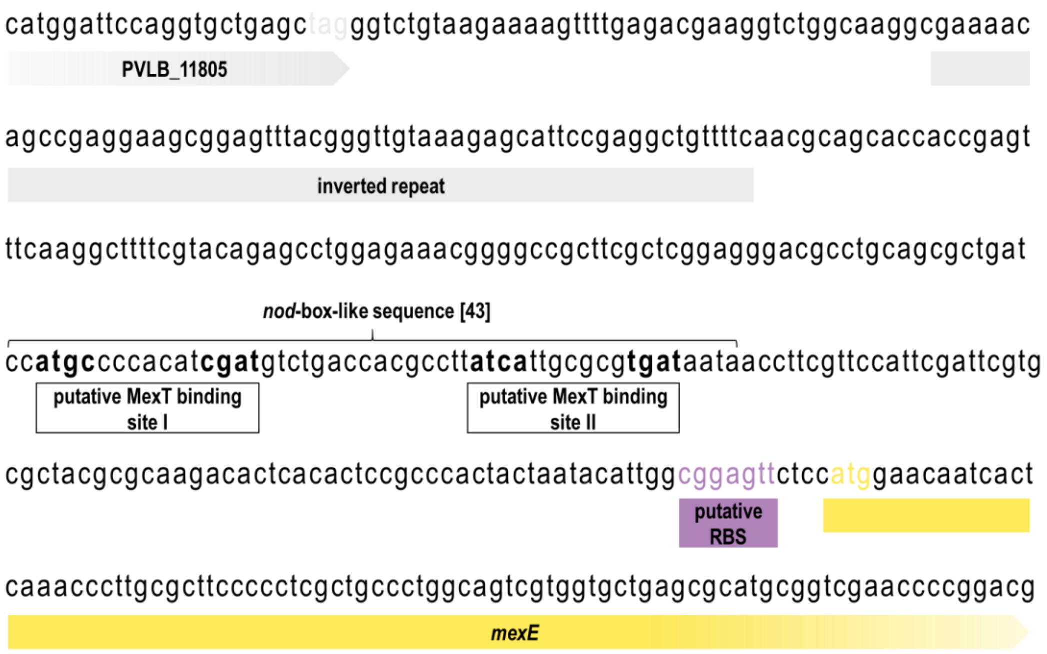

Figure S5*: Genomic context of *mexEF-oprN* in *P. taiwanensis* VLB120 highlighting the *nod*-box-like sequence containing two putative MexT binding sites upstream of the operon.* Annotations were made based on alignment with a previously determined consensus sequence ATCA(N)$_7$CGAT identified in *P. aeruginosa* (Kim et al., 2019).

Table S4: Overview of the growth parameters of all strains under the tested conditions in this work. The end of the lag phase was estimated based on the first increase in g-value by more than 5% between two measurements.

			no stressor						with HMF/furfural						
**strain**	**figure**	**carbon source**	**maximum g-value [a.u.]**				**elapsed time to reach maximum g-value [h]**	**end of lag phase [h]**	**stressor concentration [mM]**	**maximum g-value [a.u.]**				**elapsed time to reach maximum g-value [h]**	**end of lag phase [h]**
			replicates			***Avg.***				*replicates*			***Avg.***		
GRC1	2 and S2	40 mM glycerol<br>2 mM glucose	82.00	82.77	84.34	**83.03**	16.53	4	8	76.3	77	77.3	**76.88**	23.55	10.53
GRC1 ROX	2 and S2	40 mM glycerol<br>2 mM glucose	85.07	79.91	79.01	**81.33**	17.53	4	8	25.39	23.18	24.02	**24.20**	no growth	no growth
A7.1	2 and S2	40 mM glycerol<br>2 mM glucose	86.85	81.41	81.39	**83.22**	17.03	4	8	80.53	72.66	72.10	**75.10**	26.05	8
A7.2	2 and S2	40 mM glycerol<br>2 mM glucose	83.15	83.79	82.64	**83.20**	17.03	4	8	72.17	74.17	75.78	**74.04**	26.05	9.05
A8.1	2 and S2	40 mM glycerol<br>2 mM glucose	84.75	79.98	79.39	**81.37**	18.03	5	8	73.2	68.4	69.6	**70.38**	30.57	8.51
A8.2	2 and S2	40 mM glycerol<br>2 mM glucose	80.68	80.57	80.71	**80.65**	18.53	5	8	70.20	70.72	71.26	**70.73**	29.07	8
B7.1	2 and S2	40 mM glycerol<br>2 mM glucose	83.12	81.20	81.20	**81.84**	17.53	4	8	72.06	73.85	71.99	**72.63**	32.57	11.53
B7.2	2 and S2	40 mM glycerol<br>2 mM glucose	80.44	79.22	85.68	**81.78**	16.53	3	8	27.87	27.95	27.49	**27.77**	no growth	no growth
B8.1	2 and S2	40 mM glycerol<br>2 mM glucose	78.96	80.08	78.22	**79.08**	19.53	6	8	70.06	71.09	71.21	**70.79**	31.57	11.03
B8.2	2 and S2	40 mM glycerol<br>2 mM glucose	77.81	76.83	82.61	**79.08**	18.53	4.5	8	69.26	70.02	73.15	**70.81**	29.07	8
C7.1	2 and S2	40 mM glycerol<br>2 mM glucose	83.64	79.14	78.53	**80.44**	17.53	4.5	8	23.87	23.87	24.95	**24.23**	no growth	no growth
C7.2	2 and S2	40 mM glycerol<br>2 mM glucose	-	-	-	**-**	-	-	8	-	-	**-**	**-**	-	-
C8.1	2 and S2	40 mM glycerol<br>2 mM glucose	82.72	79.86	79.26	**80.61**	18.03	5	8	74.10	69.67	70.17	**71.31**	30.57	9.53
C8.2	2 and S2	40 mM glycerol<br>2 mM glucose	80.32	80.34	78.60	**79.75**	16.53	3.5	8	70.57	71.08	70.08	**70.58**	34.6	7
D7.1	2 and S2	40 mM glycerol<br>2 mM glucose	79.97	79.87	79.16	**79.67**	17.53	4.5	8	74.31	73.99	73.37	**73.89**	26.05	7.5
D7.2	2 and S2	40 mM glycerol<br>2 mM glucose	77.59	78.01	82.54	**79.38**	19.53	6	8	70.23	69.36	73.83	**71.14**	29.07	9.05

D8.1	2 and S2	40 mM glycerol<br>2 mM glucose	75.64	77.51	77.75	**76.96**	18.53	4	8	67.27	69.96	69.05	**68.76**	29.07	8
D8.2	2 and S2	40 mM glycerol<br>2 mM glucose	77.68	75.53	82.55	**78.58**	18.53	5	8	69.75	69.11	72.81	**70.55**	29.57	8
E7.1	2 and S2	40 mM glycerol<br>2 mM glucose	83.68	79.26	79.65	**80.86**	17.53	4.5	8	71.70	68.90	69.43	**70.01**	31.57	8.52
E7.2	2 and S2	40 mM glycerol<br>2 mM glucose	80.65	79.71	81.49	**80.62**	17.53	4.5	8	69.00	72.13	72.82	**71.31**	26.05	8
E8.1	2 and S2	40 mM glycerol<br>2 mM glucose	83.34	78.92	77.59	**79.95**	18.53	5	8	25.95	24.02	25.02	**25.00**	no growth	no growth
E8.2	2 and S2	40 mM glycerol<br>2 mM glucose	81.41	81.15	80.38	**80.98**	19.03	6	8	71.05	71.65	70.71	**71.14**	29.57	9.53
F7.1	2 and S2	40 mM glycerol<br>2 mM glucose	79.58	79.11	79.97	**79.55**	18.03	4.5	8	70.14	70.99	70.31	**70.48**	31.56	8
F7.2	2 and S2	40 mM glycerol<br>2 mM glucose	77.73	76.76	82.58	**79.02**	19.03	4.5	8	71.12	69.00	74.18	**71.43**	29.07	8.52
F8.1	2 and S2	40 mM glycerol<br>2 mM glucose	78.47	80.44	78.63	**79.18**	19.03	5.5	8	71.93	70.21	70.25	**70.80**	29.07	8.52
F8.2	2 and S2	40 mM glycerol<br>2 mM glucose	78.21	75.72	82.61	**78.85**	21.53	6.5	8	69.02	67.95	73.92	**70.30**	34.6	10.53
GRC1	2	20 mM glucose	99.7	100	103	**100.97**	9.58	2.48	10	94.2	96	98.4	**96.19**	13.08	7.58
GRC1 ROX	2	20 mM glucose	92.4	93.3	95.5	**93.72**	10.58	3.5	10	33.7	33.9	30.8	**32.79**	no growth	no growth
A7.1	2	20 mM glucose	91.7	92.6	92.9	**92.39**	9.58	1.5	10	83.9	85.1	84.3	**84.44**	19.08	12.08
E7.2	2	20 mM glucose	96.1	97.3	99.3	**97.59**	10.08	2.48	10	86.9	87.2	89.4	**87.85**	19.08	9.58
F8.1	2	20 mM glucose	99	97.5	96	**97.48**	13.08	6.05	10	88.3	88.1	86.8	**87.73**	22.62	13.08
GRC1	3	40 mM glycerol<br>2 mM glucose	100	101	99.7	**100.25**	15.03	3.53	8	96.8	96.4	88.3	**93.81**	20.07	9.03
GRC1 ROX	3	40 mM glycerol<br>2 mM glucose	93.5	93.7	100	**95.75**	15.03	3.03	8	30.2	30.9	29.3	**30.14**	no growth	no growth
F8.1	3	40 mM glycerol<br>2 mM glucose	95.1	101	97.7	**97.82**	16.53	4.03	8	92.1	92.6	-	**92.35**	24.57	7.53
GRC1 ROX<br>*mexT*^{G231E}	3	40 mM glycerol<br>2 mM glucose	100	97.1	101	**99.39**	16.03	3.53	8	92.5	91.5	91.1	**91.74**	24.57	6.53
GRC1 ROX<br>Δ*mexT*	3	40 mM glycerol<br>2 mM glucose	98.3	101	98.8	**99.44**	16.03	4.03	8	87.2	94.6	94.6	**92.13**	23.07	6.53
GRC1	4A	40 mM glycerol<br>2 mM glucose	100	98.8	99.2	**99.38**	16.03	2.51	8	100	99.5	92.4	**97.45**	16.52	4.01
GRC1<br>Δ*mexT*	4A	40 mM glycerol<br>2 mM glucose	98.3	98	98.9	**98.42**	16.52	2.02	8	96.1	98.2	92.6	**95.63**	15.02	3.02

GRC1	4A	40 mM glycerol<br>2 mM glucose	100	98.8	99.2	**99.38**	16.03	2.51	20	28.2	26	25.8	**26.65**	no growth	no growth
GRC1<br>Δ*mexT*	4A	40 mM glycerol<br>2 mM glucose	98.3	98	98.9	**98.42**	16.52	2.02	20	88.1	95.5	88.7	**90.75**	40.07	27.57
GRC1	4A	40 mM glycerol<br>2 mM glucose	100	98.8	99.2	**99.38**	16.03	2.51	20 (furfural)	85.6	93.2	80.9	**86.57**	23.57	11.53
GRC1<br>Δ*mexT*	4A	40 mM glycerol<br>2 mM glucose	98.3	98	98.9	**98.42**	16.52	2.02	20 (furfural)	86.2	88.9	90.4	**88.52**	21.57	10.03
GRC1	4C	40 mM glycerol<br>2 mM glucose	90.2	95.4	96	**93.86**	13.58	1.05	40	35.5	35.7	36.3	**35.85**	no growth	no growth
GRC1<br>Δ*mexT*	4C	40 mM glycerol<br>2 mM glucose	90.6	95.6	97.3	**94.52**	13.58	2	40	78.8	77	81.1	**78.95**	47.13	33.1
BOX-C1	4C	40 mM glycerol<br>2 mM glucose	92	89.1	92.4	**91.18**	14.08	2	40	34.5	34.2	32.9	**33.85**	no growth	no growth
BOX-C1<br>Δ*mexT*	4C	40 mM glycerol<br>2 mM glucose	95.7	95.9	90.3	**93.96**	14.08	1.48	40	80.3	79.2	76.8	**78.79**	47.13	31.63
BOX-C2	4C	40 mM glycerol<br>2 mM glucose	94.9	97.4	98.9	**97.04**	15.08	2	40	79.1	79.5	75.5	**78.04**	65.63	45.13
BOX-C2<br>Δ*mexT*	4C	40 mM glycerol<br>2 mM glucose	94	96	96.9	**95.64**	15.08	3.02	40	79	78	78.8	**78.60**	55.13	37.63
BOX-P2	4C	40 mM glycerol<br>2 mM glucose	95.7	94.5	90	**93.40**	14.58	2	40	68.3	71.3	70.7	**70.08**	42.63	n. a.
BOX-P2<br>Δ*mexT*	4C	40 mM glycerol<br>2 mM glucose	96.3	95.8	88.4	**93.52**	14.08	2	40	77.1	70.1	73.1	**73.41**	32.62	17.08
BOX-C1P2	4C	40 mM glycerol<br>2 mM glucose	92.9	95.5	94.9	**94.39**	14.08	2	40	80.4	77.1	-	**78.73**	41.13	24.58
BOX-C1P2<br>Δ*mexT*	4C	40 mM glycerol<br>2 mM glucose	93.7	96.5	95.2	**95.12**	14.08	2	40	77.2	76.1	72	**75.12**	32.62	20.08
BOX-C2P2	4C	40 mM glycerol<br>2 mM glucose	94.2	94.4	93.4	**94.02**	14.58	1.48	40	76.9	80.1	74.4	**77.14**	57.13	20.58
BOX-C2P2<br>Δ*mexT*	4C	40 mM glycerol<br>2 mM glucose	93.4	94	93.7	**93.71**	15.08	2	40	75.6	76.9	79.3	**77.26**	32.62	15.83
GRC1 ROX	5	40 mM glycerol<br>2 mM glucose	95.9	94.4	95.3	**95.17**	14.02	1.5	8	35.9	36.7	35.7	**36.07**	no growth	no growth
GRC1 ROX<br>Δ*mexT*	5	40 mM glycerol<br>2 mM glucose	95.8	95.5	92.2	**94.50**	15.52	2.5	8	89.7	89.2	88.9	**89.27**	21.52	3.52
GRC1 ROX<br>Δ*mexEF-oprN*	5	40 mM glycerol<br>2 mM glucose	96.6	94.8	95.9	**95.78**	15.52	2	8	88.1	86.6	87.1	**87.26**	22.02	3.52
GRC1 ROX<br>Δ*mexT*<br>Δ*mexEF-oprN*	5	40 mM glycerol<br>2 mM glucose	95.2	95.8	93.9	**94.98**	14.52	2	8	89.7	90.7	89.3	**89.92**	21.02	3.52

References

Ditta, G., Stanfield, S., Corbin, D., Helinski, D. R., 1980. Broad Host Range DNA Cloning System for Gram-Negative Bacteria - Construction of a Gene Bank of Rhizobium-Meliloti. P Natl Acad Sci-Biol. 77, 7347-7351.

Figurski, D. H., Helinski, D. R., 1979. Replication of an Origin-Containing Derivative of Plasmid Rk2 Dependent on a Plasmid Function Provided in Trans. P Natl Acad Sci USA. 76, 1648-1652.

Kim, S., Kim, S. H., Ahn, J., Jo, I., Lee, Z. W., Choi, S. H., Ha, N. C., 2019. Crystal Structure of the Regulatory Domain of MexT, a Transcriptional Activator of the MexEF-OprN Efflux Pump in *Pseudomonas aeruginosa*. Molecules and Cells. 42, 850-857.

Lechtenberg, T., Wynands, B., Wierckx, N., 2024. Engineering 5-hydroxymethylfurfural (HMF) oxidation in *Pseudomonas* boosts tolerance and accelerates 2,5-furandicarboxylic acid (FDCA) production. Metabolic Engineering. 81, 262-272.

Martinez-Garcia, E., de Lorenzo, V., 2011. Engineering multiple genomic deletions in Gram-negative bacteria: analysis of the multi-resistant antibiotic profile of *Pseudomonas putida* KT2440. Environmental Microbiology. 13, 2702-2716.

Volke, D. C., Friis, L., Wirth, N. T., Turlin, J., Nikel, P. I., 2020. Synthetic control of plasmid replication enables target- and self-curing of vectors and expedites genome engineering of *Pseudomonas putida*. Metab Eng Commun. 10, e00126.

Wynands, B., Otto, M., Runge, N., Preckel, S., Polen, T., Blank, L. M., Wierckx, N., 2019. Streamlined *Pseudomonas taiwanensis* VLB120 Chassis Strains with Improved Bioprocess Features. ACS Synthetic Biology. 8, 2036-2050.
